# Rescue Therapy With Continuous Intravenous Anakinra Infusion and Plasma Exchange in Refractory Cytokine Storm of Systemic JIA: A Case Report

**DOI:** 10.1155/crpe/1075899

**Published:** 2025-12-15

**Authors:** Ruqaiya Al Jashmi, Aza Al Sawafi, Safiya Al Abrawi

**Affiliations:** ^1^ Paediatric Rheumatology Unit, Child Health Department, Royal Hospital, Muscat, Oman, moh.gov.om

## Abstract

**Introduction:**

Macrophage activation syndrome (MAS) is one of the most common fatal complications of inflammatory diseases in children. In recent studies, Interleukin 1 inhibitors have been shown to be effective in suppressing the cytokine storm in MAS. There is limited literature describing the role of plasmapheresis in the cytokine storm syndromes as in MAS.

**Case Description:**

A six‐year‐old boy presented with a 1‐month history of daily fever, arthritis, cervical lymphadenopathy, skin rash, and elevated inflammatory markers. After multidisciplinary evaluation, he was diagnosed with systemic onset Juvenile Idiopathic Arthritis (sJIA) and started on IV methylprednisolone and SC anakinra. He later developed status epilepticus, required intubation, and progressed to multiorgan failure, needing inotropes and dialysis. Following a brief afebrile period, he worsened again, and labs confirmed MAS relapse with ferritin levels peaking at 26,000. Escalated treatment included switching to IV continuous anakinra, adding ciclosporin, and starting plasmapheresis. After three sessions of plasma exchange, he significantly improved, was extubated, and weaned off inotropes. However, attempts to switch anakinra back to SC failed twice, with recurrence of fever, rash, and elevated ferritin. He remained in PICU on IV continuous anakinra for over 3 weeks. When ferritin dropped to 900, an IV bolus regimen was trialed for a week, followed by a successful switch to SC anakinra. The child was discharged home asymptomatic with normalized labs.

**Conclusion:**

Anakinra has been used off‐label in critically ill patients with MAS, thrombocytopenia, subcutaneous edema, and neurological dysfunction. Tapering from intravenous Anakinra to subcutaneous was challenging. This case taught us that tapering must begin at the right time to avoid hiccups or unfavorable outcomes. As reported in a few studies, plasmapheresis combined with immunosuppressants can reduce MAS‐associated mortality by rapidly removing cytokines from the body.

## 1. Introduction

Systemic onset juvenile idiopathic arthritis (sJIA) is a subtype of Juvenile Idiopathic Arthritis based on the International League of Associations for Rheumatology (ILAR) classification criteria. It is diagnosed if there is arthritis in one or more joints with, or preceded by, fever of at least 2‐week duration. Signs or symptoms must have been documented daily for at least 3 days and accompanied by one or more of the following: evanescent rash, generalized lymphadenopathy, hepato/splenomegaly, and serositis [1, 2]. The diagnosis of systemic JIA, previously considered to be an autoimmune disease, is now more often classified as an autoinflammatory disease because the genetic abnormalities in the disease make it an autoinflammatory disease. A number of components have been demonstrated to be involved, including the major histocompatibility complex (MHC) and the innate immune system, such as natural killer (NK) cells, polymorphonuclear neutrophils (PMNs), and macrophages (MP), as well as Interleukin 1 (IL‐1), 6 (IL‐6), and 18 (IL‐18) [3].

MAS is considered as secondary hemophagocytic lymphohistiocytosis (sHLH), one of the most common but life‐threatening complications of autoimmune inflammatory rheumatic diseases such as sJIA, adult‐onset Still’s disease (AOSD), and systemic lupus erythematosus (SLE) [4, 5]. A nonremitting high fever, hyperferritinemia, hepatosplenomegaly, hypofibrinogenemia, and hypertriglyceridemia, as well as abnormalities of the hematological/hepatic system are all common features of this condition. The principal pathophysiological determinant of this syndrome is the aberrant activation of macrophages and T lymphocytes, which can lead to an inflammatory cytokine storm [6, 7]. The mortality rate of MAS in children varies between 8% and 23% depending on factors such as underlying disease, severity at diagnosis, and how quickly treatment is initiated [8].

Macrophages activation syndrome is a form of cytokine storm syndrome (CSS), one of the most common immune diseases characterized by constitutional symptoms, generalized inflammation, and multiorgan dysfunction that may progress to multiorgan failure without proper treatment [9, 10]. CSS is not well known among physicians, resulting in late recognition and delayed treatment. Cytokine storm reveals a variety of laboratory findings, influenced by the underlying cause.

## 2. The Case Description

**Figure 1:**
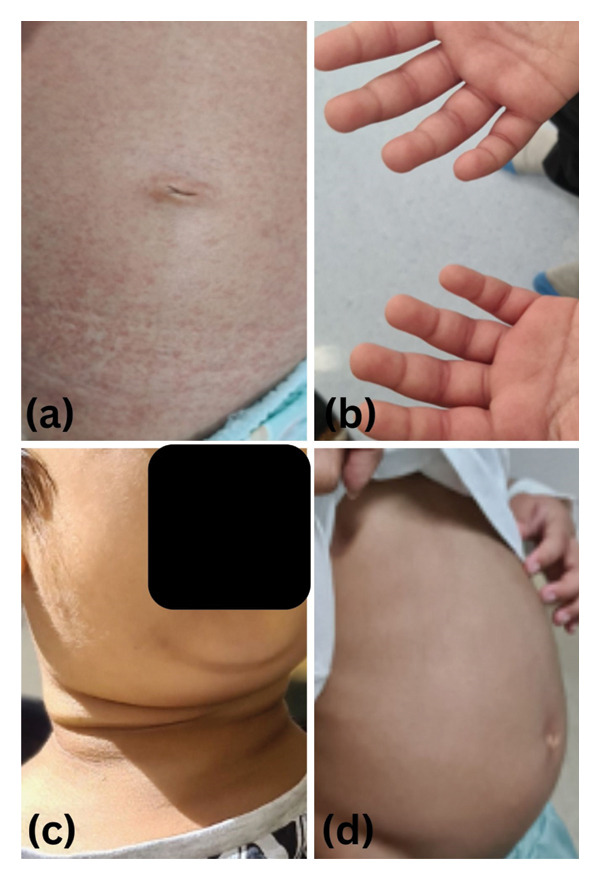
(a) The evanescent erythematous skin rash appearing with the fever spike. (b) Arthritis of the metacarpal (MCP) joints and proximal interphalangeal (PIP) joints and distal interphalangeal (DIP) joints of both hands. (c) Cervical and submandibular lymph nodes enlargement. (d) Clinical abdominal distension reflects the clinical finding of hepatosplenomegaly.

A previously healthy six‐year‐old boy presented to the Emergency Department at a tertiary hospital in Oman with a 1‐month history of a daily documented continuous fever, multiple arthritis in small and large joints, evanescent skin rash, cervical lymphadenopathy, and hepatosplenomegaly (Figures 1(a), 1(b), 1(c), 1(d)). The patient was admitted under the general pediatric team’s care for evaluation of pyrexia of unknown origin. In the first instance, the patient has been reviewed by three main teams including (infectious diseases, rheumatology, and haemato‐oncology). His labs on admission were as follows: Hb: 8.3 g/dL, platelets: 390 × 10 ∗ 9/L, WBC: 17.1 × 10 ∗ 9/L (ANC: 13.8, lymph: 2.3), ESR: 91 mml/h, CRP: 108 mg/L, ferritin: 1825 μg/L, LDH: 402 iμ/L, ALT 14 iμ/L, AST 19 iμ/L, triglycerides: 1.5, and fibrinogen 4.68.

Upon ruling out infectious and oncologic causes for the child’s symptoms, the child was diagnosed with systemic JIA. A course of intravenous methylprednisolone (IVMP) at 30 mg/kg per day was started for the patient, and anakinra was given to him subcutaneously (SC) at 3 mg/kg per day. Despite the fact that his fever had subsided after treatment, he fell into a state of status epilepticus on the third day of treatment and had to be intubated and moved to a Pediatric Intensive Care Unit (PICU). Radiological imaging of the brain (MRI, MRA, and MRV) revealed reversible, generalized atrophy, and his laboratory results confirmed macrophage activation syndrome (MAS) with a ferritin level of 26,195 μg/L, LDH: 1681 iu/L, D‐dimer: 33.34, fibrinogen: 1.49, triglycerides: 5.84, AST: 226 iu/L, ALT: 287 iu/L, ESR of 67 mml/h, CRP of 22 mg/L, HB of 8.1 g/dL, platelets of 127 × 10 ∗ 9/L, WBC of 7.5 × 10 ∗ 9/L, and ANC of 1.9 × 10 ∗ 9/L, and IL‐2 receptor concentration was over 20 ng/mL. Antiepileptic therapy was initiated, and escalation of MAS‐directed treatment was undertaken on the same day as clinical deterioration (Day 3 of treatment). The subcutaneous anakinra dose was increased to 6 mg/kg/day, and intravenous (IV) ciclosporin was added at a dose of 0.5 mg/kg twice daily (BID).

**Figure 2:**
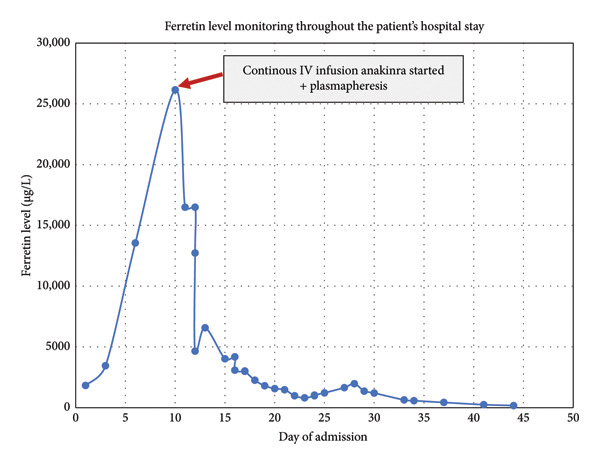
Illustration of ferritin level throughout hospital stay and the dramatic improvement in relation to initiation of IV anakinra + plasmapharesis.

In spite of the escalation in therapy, the child continued to have fever spikes. His septic workup was negative, and he was treated empirically with three antimicrobials including meropenem, vancomycin, and acyclovir). As of Day 4, the child’s condition had worsened to the point that he was on five inotropic supports, anuric, and required renal dialysis with continuous renal replacement therapy (CRRT). As a result, plasmapheresis was initiated, anakinra was changed to continuous IV infusion at a rate of 8 mg/kg/day, IVMP was changed to IV dexamethasone according to HLH protocol, and the dosage of IV ciclosporin was increased to 1 mg/kg per dose BID. A dramatic improvement was observed following daily sessions of plasmapheresis for three consecutive days, continuous infusion of IV Anakinra, IV ciclosporin, and IV dexamethasone. On the fifth day after his admission to the PICU, he was successfully extubated. There was a significant improvement in his MAS lab results with a ferritin level drop to 2997 μg/L (Figure 2).

An attempt to change IV anakinra from continuous infusion to IV BID boluses at the same total daily dose has been made; however, the child started to have multiple fever spikes. Therefore, we went back to continuous IV infusions of anakinra and fever resolved and MAS labs improved. A week later, when the ferritin level became 1400 μg/L, another trial was conducted to overlap SC anakinra with IV continuous anakinra infusion at a total daily dose of 12 mg/kg/day (6 mg/kg as IV and 6 mg/kg as SC) and again fever with skin rash appeared on the following day. Decision was made to stop SC anakinra and to increase the rate of IV anakinra infusion to 8 mg/kg/day. The child continued to have a low‐grade fever for a couple of days with the appearance of erythematous rashes, and his ferritin level rose up again to 1600 μg/L. The treatment included the addition of IV methylprednisolone (10 mg/kg/day) on top of IV dexamethasone (5 mg/m^2^/day administered in two divided doses).

As soon as ferritin reached 900, IV anakinra continuous infusion was stopped and a bolus of 6 mg/kg was given an hour later and then repeated 12 h later. The child continued on IV anakinra 100 mg BID (6 mg/kg/dose) for a week before switching to SC at the same dose. Ciclosporin was switched to oral at 5 mg/kg/dose BID, and IVMP at an equivalent dose (40 mg IV once daily) was changed to oral prednisolone at 2 mg/kg/day. Upon hospital discharge, the MAS labs were completely normal with ferritin 127 μg/L and he was seen again in the outpatient clinic four days later in complete clinical and laboratory remission. The child remained neurologically stable, allowing for discontinuation of antiepileptic medications after 3 months. A follow‐up brain MRI subsequently demonstrated normal findings. He has been followed up regularly in outpatient clinic since March 2023 and for two years, with complete clinical and biochemical remission. During the first 12 months, he received daily subcutaneous anakinra (100 mg), followed by 6 months of subcutaneous tocizullimab (IL‐6 blocker). He has been off treatment since September 2024 and remains completely asymptomatic.

## 3. Discussion

Delayed presentation to healthcare services can lead to severe and potentially preventable complications. In the present case, the patient’s late arrival at the hospital resulted in delayed initiation of appropriate therapy. Despite timely commencement of treatment upon admission, the clinical course was complicated by the subsequent development of MAS and CSS. Early recognition of the evolving cytokine storm allowed for prompt escalation of therapy, which was crucial in mitigating further deterioration.

This case represents our institution’s first experience utilizing continuous IV anakinra infusion, which resulted in a favorable outcome despite a complex intensive care unit (ICU) course. The patient’s eventual recovery and discharge in good overall health underscore the effectiveness of a multimodal therapeutic strategy combining continuous IV anakinra, plasma exchange, ciclosporin, and glucocorticoids.

Subcutaneous administration of anakinra may be inadequate for achieving therapeutic serum concentrations in critically ill patients, as highlighted by this case. Conditions such as subcutaneous edema, impaired tissue perfusion, hemodynamic instability, and severe thrombocytopenia can all compromise subcutaneous drug absorption. Therefore, IV administration is preferred during the acute phase of illness, as premature transition to subcutaneous dosing may lead to subtherapeutic exposure [11].

In severe MAS or cytokine storm, several reports have documented the use of high‐dose IV anakinra, including continuous infusion regimens dosed in mg/kg/day. Such regimens frequently exceed the practical limits of subcutaneous administration, which would otherwise require multiple injection sites or increased dosing frequency (once or twice daily) to achieve comparable therapeutic levels [11]. Continuous IV infusion enables rapid attainment of high serum concentrations and sustains stable therapeutic exposure, whereas subcutaneous administration is characterized by slower absorption, lower peak plasma concentrations, and longer apparent half‐life, with a reported bioavailability of approximately 80%–92% [12, 13].

Transitioning from IV to subcutaneous anakinra presents additional clinical challenges. Current evidence is largely restricted to case series and small cohort studies describing diverse approaches—such as alternate‐day dosing, gradual dose reduction, or interval extension—with no standardized or validated protocols. To date, there are no large randomized controlled trials or consensus guidelines to inform optimal tapering or switching strategies. Consequently, treatment transitions must be individualized according to patient response and clinical judgment, as exemplified in this case [14].

## 4. Conclusion

This case underscores the critical importance of early recognition and aggressive management of CSSs in pediatric patients. It also supports the use of continuous IV anakinra as a viable therapeutic option when subcutaneous absorption is likely to be unreliable. The administration of anakinra via the IV route remains an off‐label practice [12–16], and currently, no standardized protocols or evidence‐based guidelines exist to guide the transition from IV to subcutaneous administration.

In this case, determining an effective tapering strategy for anakinra posed a significant clinical challenge. Our experience highlights the importance of initiating tapering only after clear evidence of clinical stabilization, using both clinical parameters and inflammatory markers to guide timing. Such an approach helps prevent disease flare, treatment interruption, and other adverse outcomes during the transition phase.

Furthermore, emerging evidence suggests that plasmapheresis may serve as a beneficial adjunct in the management of MAS. By promoting the clearance of circulating cytokines and inflammatory mediators, plasmapheresis may reduce mortality when used alongside optimal immunosuppressive therapy [17, 18]. This case reinforces the potential value of a multimodal therapeutic strategy—combining targeted cytokine inhibition, immunosuppression, and plasma exchange—in achieving favorable outcomes in severe or refractory MAS.

## Consent

All the patient and parents allowed personal data processing, and informed consent was obtained from parents included in the case report.

## Disclosure

This abstract has been presented in the PRes 2023 as poster and published in Proceedings of the 29th European Pediatric Rheumatology Congress with the following title: Combination Therapy of Plasmapheresis with Continuous Intravenous Anakinra Infusion to Calm the Cytokine Storm in Macrophages Activation Syndrome Secondary to Systemic Juvenile Idiopathic Arthritis.

## Conflicts of Interest

The authors declare no conflicts of interest.
